# Development and *In Vitro*/*Ex Vivo* Evaluation of Lecithin-Based Deformable Transfersomes and Transfersome-Based Gels for Combined Dermal Delivery of Meloxicam and Dexamethasone

**DOI:** 10.1155/2022/8170318

**Published:** 2022-11-29

**Authors:** Muhammad Imran Khan, Samiya Yaqoob, Asadullah Madni, Muhammad Furqan Akhtar, Muhammad Farhan Sohail, Ammara Saleem, Nayab Tahir, Kashif-ur-Rehman Khan, Omer Salman Qureshi

**Affiliations:** ^1^Riphah Institute of Pharmaceutical Sciences (RIPS), Riphah International University, Lahore Campus, 54000 Lahore, Pakistan; ^2^Department of Pharmaceutics, Faculty of Pharmacy, The Islamia University of Bahawalpur, Bahawalpur 63100, Pakistan; ^3^Department of Pharmacology, Faculty of Pharmaceutical Sciences, Government College University Faisalabad, Pakistan; ^4^College of Pharmacy, University of Sargodha, Sargodha, Pakistan; ^5^Department of Pharmaceutical Chemistry, Faculty of Pharmacy, The Islamia University of Bahawalpur, Bahawalpur 63100, Pakistan; ^6^Forman Christian College University, Lahore, Pakistan

## Abstract

Transfersomes (TFS) are the promising carriers for transdermal delivery of various low and high molecular weight drugs, owing to their self-regulating and self-optimizing nature. Herein, we report synthesis and characterization of TFS loaded with meloxicam (MLX), an NSAID, and dexamethasone (DEX), a steroid, for simultaneous transdermal delivery. The different formulations of TFS containing varying amounts of lecithin, Span 80, and Tween 80 (TFS-1 to TFS-6) were successfully prepared by thin-film hydration method. The size of ranged between 248 and 273 nm, zeta potential values covering from –62.6 to –69.5 mV, polydispersity index (PDI) values in between 0.329 and 0.526, and entrapment efficiency of MLX and DEX ranged between 63-96% and 48-81%, respectively. Release experiments at pH 7.4 demonstrated higher cumulative drug release attained with Tween 80 compared to Span 80-based TFS. The scanning electron microscopy (SEM) of selected formulations -1 and TFS-3 revealed spherical shape of vesicles. Furthermore, three optimized transfersomal formulations (based on entrapment efficiency, TFS-1, TFS-3, and TFS-5) were incorporated into carbopol-940 gels coded as TF-G1, TF-G3, and TF-G5. These transfersomal gels were subjected to pH, spreadability, viscosity, homogeneity, skin irritation, *in vitro* drug release, and *ex vivo* skin permeation studies, and the results were compared with plain (nontransfersomal) gel having MLX and DEX. TFS released 71.72% to 81.87% MLX in 12 h; whereas, DEX release was quantified as 74.72% to 83.72% in same time. Nevertheless, TF-based gels showed slower drug release; 51.54% to 59.60% for MLX and 48.98% to 61.23% for DEX. The TF-G systems showed 85.87% permeation of MLX (TF-G1), 68.15% (TF-G3), and 68.94% (TF-G5); whereas, 78.59%, 70.54%, and 75.97% of DEX was permeated by TF-G1, TF-G3, and TF-G5, respectively. Kinetic modeling of release and permeation data indicated to follow Korsmeyer-Peppas model showing diffusion diffusion-based drug moment. Conversely, plain gel influx was found mere 26.18% and 22.94% for MLX and DEX, respectively. These results suggest that TF-G loaded with MLX and DEX can be proposed as an alternate drug carriers for improved transdermal flux that will certainly increase therapeutic outcomes.

## 1. Introduction

Nonsteroidal anti-inflammatory drugs (NSAIDs) are among the most frequently prescribed drugs due to their analgesic, antipyretic, and anti-inflammatory effects. A range of inflammatory diseases such as rheumatoid arthritis, osteoarthritis, and ankylosing spondylitis require the use of NSAIDs for longer times [1]. NSAIDs exert their anti-inflammatory action by inhibiting the cyclooxygenase enzymes with a consequent reduction in the prostaglandin level [2]. These prostaglandins are crucial to prevent the stomach lining from the corrosive action of the stomach acid. Thus, stomach disturbance is the major adverse effect associated with continuous oral therapy of the NSAIDs. Other systemic adverse effects include platelet aggregation inhibition, anemia, kidney damage, and cardiac degradation [3]. Therefore, the transdermal delivery of NSAIDs for the local action is a desirable approach for the optimal management of inflammatory conditions of skin as an alternative parenteral or oral therapy. Given this, various conventional dosage forms have been developed like transdermal gels, ointments, and creams for their longer contact time with the skin surface and releasing the therapeutic moiety to be absorbed via the skin [4]. However, the skin is not freely permeable to all drugs, and these dosage forms are suitable only for those drugs that can easily permeate the stratum corneum (SC) of the skin. Several formulation strategies have been adopted to enhance the delivery of limited permeable drugs against the SC layer including the incorporation of a chemical permeation enhancer, iontophoresis, electrophoresis, and microneedle patches [5]. Recently, topical delivery of therapeutic moities incorporated into lipid-based carriers like liposomes, niosomes, ethosomes, and transfersomes have attained huge attention [6].

The vesicular carrier system can entrap drug molecules by acting as a drug carrier and increase the permeation of drugs into the stratum corneum. As far as topical vesicular systems are concerned, unfortunately, the first-generation nanovesicular systems including liposomes and niosomes were accompanied with drawbacks, particularly weak stratum corneum (SC) penetration because of their inflexible nature [7]. Among all other vesicular carriers, ultradeformable vesicular drug carrier system “Transfersomes”(TFS) have been considered as surrogate vehicles for transdermal drug delivery of a wide range of hydrophilic as well as hydrophobic drug molecules. TFS are complex aggregates of highly deformable and stress-responsive vesicles. TFS are the first-generation deformable vesicles developed for the first time by Cevc and Blume [8]. TFS could be loaded with a variety of low molecular weight drugs as well as with high molecular weight drugs [9]. In this context, there are several reports mentioning the transfersomal loading of insulin [10], interferon-alpha [11], diclofenac [12], tamoxifen, hydrocortisone, estradiol, and cyclosporine [13]. Structurally, their composition includes phospholipids which form a lipid bilayer, and a surfactant such as Span, Tween, sodium cholate, or deoxycholate. The role of surfactant is that of an “edge activator” which decreases the rigidity of lipid bilayer and increase flexibility of the TFS [14]. This structure mimics cell vesicles or cells engaged in exocytosis which make these carriers more preferable for controlled and targeted drug delivery [15]. In this study, lecithin was used as main component of TFS due to its inherent characteristics such as solubilization of the substances with different physiochemical properties, biocompatibility, thermodynamic stability, thermoreversible nature, and resistance to microbial contamination [16]. As the lecithin itself provides skin protection against UV induced skin aging, it shows additional effects along with incorporated bioactive agents against skin aging [17]. A wide variety of guest molecules such as vitamins A and C, hormones, NSAIDS, peptides, amino acids, local anesthetics, and antifungal agents were reported to be effective topically when delivered by lecithin-based delivery systems [18]. Therefore, keeping in view of above advantages, lecithin was used as lipid component for fabrication of TFS.

Gels are considered promising vehicles to retain drugs or nanoparticles at the site of application. Gels also increase the residence time of the drug due to their mucoadhesive character [19]. The incorporation of TFS in carbopol-940 gel matrix in current study would have potential to increase the residence time at the inflamed body site, enabling the TFS to penetrate efficiently through the skin for a longer durations.

Therefore, this research is aimed at developing TFS loaded with meloxicam (MLX) and dexamethasone (DEX) and subsequent incorporation into carbopol-940 gel for enhanced skin permeation, which may have the ability to suppress adverse effects associated with peroral therapy and treat severe pain and inflammatory conditions effectively. TFS were characterized for entrapment efficiency (EE), vesicle size, polydispersity index (PDI), zeta potential, release profiles, and *in vitro* permeation studies. Kinetic models were applied to release data of TFS to find out best fit model and mechanism of release. TF-based gels (TF-Gs) were also evaluated for spreadibility, viscosity, homogeneity, drug release, permeation potential, and anti-inflammatory activity in induced paw edema rat model.

## 2. Materials and Methods

### 2.1. Materials

Meloxicam was gifted by Alcon Biosciences Laboratories (Lahore, Pakistan) while dexamethasone was donated by Schazoo Pharmaceutical Laboratories (Lahore, Pakistan). Methanol, chloroform, and sodium hydroxide were obtained from Sigma-Aldrich (Germany) and lecithin from Duksan Pure Chemicals Co., Ltd. (South Korea). Tween 80 was supplied by Honeywell Riedel-de Haen (Germany), Span 80 from Daejung Chemicals & Metal Co., Ltd. (Korea), and potassium phosphate from Honeywell Riedel-de Haen (Germany). Carbopol-940 was taken from BDH Chemicals (UK). Distilled water was obtained from the distillation plant of Riphah Institute of Pharmaceutical Sciences, Riphah International University, Lahore.

### 2.2. Methods

#### 2.2.1. Preparation of Transfersomes

Six different formulations of transfersomes (TFS-1-TFS-6) loaded with meloxicam (MLX) and dexamethasone (DEX) were prepared using the thin-film hydration (TFH) method reported elsewhere [20]. The composition of all the six TFS is presented in Table 1. MLX, DEX, lecithin, Tween 80, and Span 80 were accurately weighed and dissolved in a 20 mL mixture of methanol and chloroform (3 : 1 *v*/*v*). The organic phase was evaporated using a rotary evaporator (Heidolph, Germany) by heating at 60°C above the phase-transition temperature of lecithin (52°C). A thin film was formed along the wall of the flask which was dried completely in a vacuum desiccator nightly. Finally, the dried lipid thin film was hydrated with 20 mL phosphate buffer (pH 6.4) with gentle shaking for 30 min, followed by sonication for 2 h at 2-6°C in a bath sonicator. The prepared TFS (20 mL) were stored overnight at 4°C for further evaluation.

#### 2.2.2. Fourier Transform Infrared (FTIR) Spectroscopy

The FTIR spectra were obtained for pure MLX and DEX, and physical mixtures of drugs with excipients using FTIR spectrophotometer (ALPHA platinum ATR, USA). FTIR spectra of pure drugs were coded as A-1 for MLX and A-2 for DEX. Physical mixtures (1 : 1) were prepared by mixing MLX and DEX with Tween 80, Span 80, lecithin, and carbopol-940 separately and coded as B-1, B-2, B-3, and B-4. A physical mixture of all ingredients used in preparation was also observed and coded as B-5. Finally, samples were mounted on the ATR crystal of the spectrophotometer and resultant spectra were recorded at wavelength range (4000-600 cm^−1^) with a resolution of 4 cm^−1^ at ambient temperature [21].

#### 2.2.3. Determination of Entrapment Efficiency

TFS (TFS-1-TFS-6) were evaluated for entrapment efficiency (EE) by separating the free drug from TFS using the ultracentrifugation method [22]. TFS were subjected to ultracentrifugation after placing them in centrifugation tubes and centrifuged at relative centrifugal force of 21952 g for 2 hours at 4°C in the cooling centrifuge (Sigma, Germany). After centrifugation, the supernatant was withdrawn and diluted appropriately with phosphate buffer pH 6.8 and analyzed at 364 nm and 248 nm for MLX and DEX determination, respectively, using a UV spectrophotometer (Agilent USA) [23]. All the results were taken in triplicate and the following mathematical expression was used to calculate the amount of drug entrapped.

(1) $$\begin{array}{c} \%\text{Entrapment}=\frac{\text{Total drug}-\text{drug in supernatant}}{\text{Total drug}}\times 100. \end{array}$$

#### 2.2.4. Measurement of Vesicle Size, Polydispersity Index, and Zeta Potential

The dynamic light scattering (DLS) method was used for the measurement of vesicle size, polydispersity index (PDI), and zeta potential using zetasizer (Zetasizer Malvern Instruments, UK). Based on better %EE results, three optimized formulations of transfersomes, i.e., TFS-1 (containing Span 80), TFS-3 (containing Tween 80), and TFS-5 (containing both Span 80 and Tween 80), were subjected for vesicle size, PDI, and zeta potential measurements. From each optimized formulation, transfersomal suspension (1 mL) was taken and diluted with deionized water (10 mL) for measurement. Each test formulation was subjected three times for analysis [24].

#### 2.2.5. Morphology of Vesicles

The morphology of two selected optimized transfersomal formulations, i.e., TFS-1 and TFS-3, were analyzed using scanning electron microscopy (SEM). A drop from each formulation was placed on the copper grid and air-dried. After drying, all samples were visualized using SEM (FEI Nova, USA) at an accelerated voltage of 30 kV, and images were taken at different magnifications ranging from 5,000 xto 50,000 x [25].

#### 2.2.6. Release Studies of Transfersomes

The release of MLX and DEX from prepared TFS (TFS-1, TFS-2, TFS-3, TFS-4, TFS-5, and TFS-6) was evaluated using a dialysis tube (M wt. 12.000-14.000 Da) loaded with a volume (ranged 1-3 mL depending upon entrapment efficiency) equivalent to 3 mg of drugs and immersed in 100 mL phosphate buffer saline (pH 7.4) at 37°C with shaking at 100 rpm in a thermostatically shaker water bath (D3165 Hangisen, W-Germany) for 24 h. 2 mL aqueous solution containing 3 mg of pure MLX and DEX was experimented for comparison purpose [26]. Aliquots of 2 ml were taken at 0, 1, 2, 3, 4, 6, 8, 10, and 12 h time intervals and replaced with equal volume of fresh buffer and analyzed for drug content by UV spectroscopy at 362 nm and 240 nm for MLX and DEX, respectively. Experiments were repeated three times and the results were expressed as the mean values ± SD. Mechanism of drug release was determined by fitting the release profiles to equations of zero order, first order, Higuchi diffusion, and Korsmeyer-Peppas and the correlation was used as an indicator of goodness-of-fit [27].

#### 2.2.7. *Ex Vivo* Skin Permeation Studies of Transfersomes

*Ex vivo* skin permeation studies were performed through the hairless skin of rat using Franz diffusion cell [28]. The abdominal skin of rat was excised and subcutaneous fat was removed with a dull scalpel. The skin was stored in normal saline solution (pH 5.5). The excised skin was then mounted between the donor and receptor compartments of the Franz diffusion cell with the dermal side in contact with the receptor medium and the stratum corneum side facing upwards into the donor compartment. Receptor compartment of Franz diffusion cell was filled with 10 mL of freshly prepared phosphate buffer having pH 6.8 and was stirred continuously by a magnetic stirrer. The temperature in the receptor compartment was maintained at 32 ± 0.5°C to simulate the skin temperature. Then, transfersomal formulations equivalent to ~500 *μ*g of MLX and 1 mg of DEX were evenly applied on the surface of the rat skin in the donor compartment. 1 mL of sample was withdrawn from the sampling port of Franz diffusion cell at specified time intervals (0, 1, 2, 3, 4, 5, and 6 h) throughout 6 h. After each sampling, an equal volume of phosphate buffer was added to maintain the sink conditions throughout the experiment. These samples were diluted suitably with phosphate buffer and drug contents from each formulation were calculated. Content of MLX and DEX was measured using a UV-visible spectrophotometer at *λ*_max_ of 364 nm and 248 nm, respectively. These permeation studies were performed for all the six TFS [29].

#### 2.2.8. Preparation of Transfersomal Gel (TF-G) and Plain Gel (without Transfersomes)

Three optimized TF formulations (TFS-1, TFS-3, and TFS-5) were chosen for the preparation of TF-based gels (TF-G). 2 g of carbopol-940 was dispersed in 50 mL phosphate buffer pH 6.8 and left overnight at room temperature. The weighed amount of each selected TFS, equivalent to 50 mg MLX and 25 mg DEX, was incorporated into the gel with gentle stirring. The prepared gels were stored at 4-6°C and designated as TF-G1, TF-G3, and TF-G5. The plain gel (without transfersomes) was also prepared with 50 mg MLX and 25 mg DEX to compare the drug permeability profile of TF-G as well as plain gel [20].

#### 2.2.9. Evaluation of Transfersomal Gel

*(1) Measurement of pH*. The apparent pH of all three optimized gel formulations (TF-G1, TF-G3, and TF-G5) and the plain gel were measured using a digital pH meter (Hanna, USA) in triplicate at room temperature. pH meter was calibrated at two points against the standard buffers of pH 4.0 ± 0.1 and 7.0 ± 0.1 as per established criteria for calibration of pH meter prior to its use [30].

*(2) Spreadability*. Spreadability TF-G (TF-G1, TF-G3, and TF-G5) and the plain gel were determined by placing the sample between two glass slides in which the lower slide was fixed while the upper one tied to a standard weight of 25 g with the help of a hook. 1 g of each prepared TF-G was placed on separate slides and spreadability was measured by using the formulae as presented in

(2) $$\begin{array}{c} \text{s}=W\times \frac{\text{L}}{\text{t}}, \end{array}$$

where *s* is the spreadability, *W* is the weight tide to upper slide, *L* is the length of slide, and t is the time taken by slide to detach.

*(3) Viscosity Determination*. TF-G (TF-G1, TF-G3, and TF-G5) and plain gel were tested for their viscosity measurement using a viscometer (Brookfield Engineering Laboratories, USA). For the measurement of viscosity, 50 g of each prepared batch was taken in 100 mL of beaker and spindle T-95 was dipped perpendicularly into the gel up to the particular mark on the spindle. The spindle speed was set at 30 rpm and determined at room temperature for 5 minutes [31].

*(4) Homogeneity*. TF-G1, TF-G3, TF-G5, and plain gel were observed visually for homogeneity studies. Gel formulations were taken in a neat and clear beaker and inspected for the presence of any aggregated or suspended particles.

*(5) Skin Irritation Studies*. Skin irritation potential of MLX- and DEX-loaded TF-G (TF-G1, TF-G3, and TF-G5) was assessed by carrying out skin irritancy tests on Wistar Albino rats (200-300 g) [32]. These rats were allowed to live for one week before the start of the study. The dorsal surface of the rat was shaved without harming the skin surface, approximately 4 h before the experiment. The rats were segregated into four groups (*n* = 3): group I: plain gel (negative control), group II: MLX- and DEX-loaded solution, group III: aqueous solution of formalin (positive control), and group IV: transfersome-based gel loaded with MLX and DEX. The formulations (100 mg containing 0.1 mg of drugs each) were topically applied to hairless skin area (1 cm^2^). After topical application, rats were returned to their respective cages and were examined at 24, 48, and 72 h. The signs of erythema and edema were noticed. The average erythemal and edema scores were recorded based on severity caused by topical application of formulations, if there is no edema/erythema = 0, slight erythema/edema = 1, moderate erythema/edema = 2, and severe erythema/edema = 3 [33].

*(6) Release Studies of Transfersome-Based Gels*. The release of MLX and DEX from prepared TF-G (TF-G1, TF-G3, TF-G5, and TF-5) was investigated with same experimental protocol given in Section 2.2.7.

*(7) Ex Vivo Skin Permeation Studies of Transfersome-Based Gels*. *Ex vivo* drug penetration into the skin from different transfersomal gel TF-G (TF-G1, TF-G3, and TF-G5) was compared with a plain gel having the same amount of active drug ingredients by employing Franz diffusion cell. 3 g of TF-G was accurately weighed and applied on the skin of rat following same experimental protocol as illustrated in part 2.2.7.

*(8) Anti-Inflammatory Activity Studies of Transfersome-Based Gels*. Anti-inflammatory activity of TF-G (TF-G1, TF-G3, and TF-G5) was evaluated by monitoring the zone of inhibition of hind paw edema induced by the phlogistic agent, i.e., carrageenan. In the current study, 0.1 mL aliquot of 1% *w*/*v* carrageenan solution was used as a phlogistic agent. Albino rats having weight of 200-300 g were selected for the study. The animals were divided into two groups, each consisting of six rats. Group 1 was treated with the plain gel of drugs (MLX and DEX) which acted as control; whereas, group II was treated with test transfersomal-based gel (TF-G1). The animals fasted overnight and both groups were treated by applying 1 g of respective gel formulations on the left paw of each rat. The area was occluded with bandages and it was left in place for 1 h. The dressing was then removed, and the remaining gel was wiped off with cotton. 1%*w*/*v* carrageenan solution was injected into the plantar part of the left hind paw, and paw volume was measured after 1 h, 2 h, 4 h, and 5 h using the water displacement method. The percent edema produced with test samples was subtracted from the percent edema produced in the control group to obtain percent edema inhibition. Percent inhibition of edema is directly proportional to anti-inflammatory activity.

#### 2.2.10. Statistical Analysis

All measurements were made in triplicate. The data were expressed as mean values ± standard deviation. The data were analyzed by independent sample *t*-test and one way ANOVA. A value of *p* < 0.05 was considered statistically significant.

## 3. Results

### 3.1. Drug-Excipient Interaction Studies

**Figure 1:**
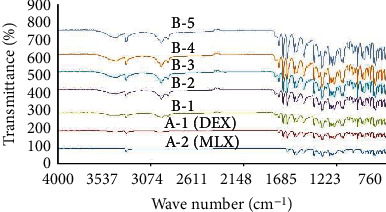
FTIR spectra of MLX (A-2), DEX (A-1), mixture of MLX+DEX+Span 80 (B-1), mixture of MLX+DEX+Tween 80 (B-2), mixture of MLX+DEX+lecithin (B-3), mixture of MLX+DEX+carbapol (B-4), and physical mixture of all ingredients (B-5).

Figure 1 shows the FTIR spectra of pure drugs MLX and DEX as well as the physical mixtures. The remarkable spectrum features of MLX include the prominent peaks that appear at 3285 cm^−1^ (for stretching vibration band of NH), 1452 cm^−1^ (for C=C stretching of aromatic ring), and 1617 cm^−1^ (for C=O stretching of amid) [34] [35], which can be observed in Figure 1. The FTIR spectrum of DEX is shown as A-1 (red colour spectrum). The characteristic peaks can be observed at 900 cm^−1^ (which confirms the presence of DEX), 1268 cm^−1^ (for starching vibration of C-F bond), and 1709 cm^−1^ (for –C=O) [36]. The same peaks appeared in other spectra of physical mixtures shown in Figure 1.

The characteristic peaks of DEX were being found in spectra of physical mixtures (B-1, B-2, B-3, B-4, and B-5) indicating compatibility of ingredients with model drugs. Similarly, characteristic peaks of MLX were found intact in physical mixtures coded as B-1, B-2, B-3, B-4, and B-5. The overall results of the study revealed chemical compatibility between model drugs MLX and DEX with vesicular ingredients (raw materials) because there were no alterations in band spectra when compared with each separate spectrum of pure drug.

### 3.2. Entrapment Efficiency Studies

The percent entrapment efficiency (%EE) of TF formulations was determined and summarized in Table 2. UV-visible spectrophotometer was used to determine entrapment efficiency and expressed in terms of mean %EE ± SD.

### 3.3. Vesicle Size, PDI, and Zeta Potential of Transfersomes

Vesicle size, PDI, and zeta potential are important physicochemical characteristics that affect the *in vitro* and *in vivo* behavior of the colloidal drug delivery systems. The average vesicle size of TFS ranged from 248 ± 3 to 273 ± 4 nm. PDI of vesicle size distribution ranged from 0.329 ± 0.03 to 0.526 ± 0.05. It is agreed upon that PDI values below 0.7 indicate monodispersed system [37]. PDI was less than 0.7 for TFS which indicated monodiperse system. Zeta potential was determined to study the surface properties of developed transfersomes because surface charge of vesicles plays an essential role in stability of formulations by creating a repulsive energy barrier to prevent aggregation [38]. The zeta potential values of transfersomes in the study were found to be in the range of −62.6 ± 2 to − 69.5 ± 5 mV imparting considerable stability to transfersomes. It is also identified that some surfactants type and their concentrations greatly affect zeta potential values. In a previous report, the net charge on the vesicle surfaces was thought to be the combination of both lipid and surfactant charge. Nevertheless, nonionic surfactant could not ionize into charging group like ionic, but can demonstrate its zeta potential; the reason might be due to molecular polarization [39]. Higher zeta potential values indicated the stability of transfersomes and minimum attractive forces that resist their agglomeration [40]; hence, more electrically stable colloid system was achieved. The negative charge was considered as favorable for stability and improved penetration of transfersomes due to similar charges [41]. The size, PDI, and zeta potential values of TF are presented in Table 2.

### 3.4. Morphology of Vesicles

**Figure 2:**
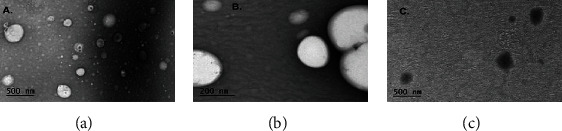
SEM images of optimized transfersomes (a) TFS-1, (b) TFS-3, and (c) TFS-5. Magnification up to 5000 x. Scale bars are given on images.

The optimized formulation TFS (TFS-1, TFS-3, and TF-5) were subjected to morphological evaluation using SEM. SEM images of vesicles revealed distinct almost spherical vesicles with a clear outline. The images showed that developed vesicles are of colloidal dimension. The outline and core of vesicles confirmed vesicular characteristics indicating the integrity of closed structures. The vesicles are smaller unilamellar vesicles with a more homogenous size distribution. SEM images are shown in Figure 2.

### 3.5. Drug Release Studies from Transfersomes

**Figure 3:**
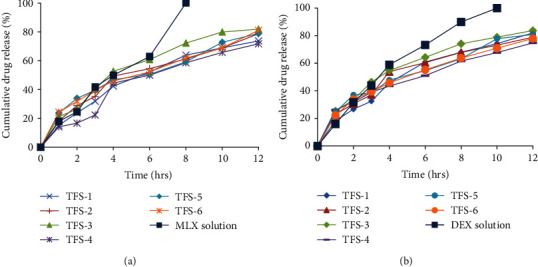
Release profiles of MLX (a) and DEX (b) from transfersomes at pH 7.4.

Drug release patterns of pure drug solutions and TFS at physiological pH 7.4 are represented in Figure 3. MLX and DEX release from designed TFS was considerably lessen as compared to the drug solutions. From the release graphs, 62.89% of MLX was released from solution with in first 6 h and 100% within 8 h. Providently, TFS-1, TFS-2, TFS-3, TFS-4, TFS-5, and TFS-6 released 73.70%, 78.70%, 81.87%, 71.72%, 78.89%, and 81.08%, respectively, after 12 hours. The order of release was TFS − 3 > TFS − 6 > TFS − 5 > TFS − 2 > TFS − 1 > TFS − 4 for MLX. On the other hand, DEX release was observed to be 81.97%, 78.89%, 83.72%, 74.72%, 80.89%, and 77.98% for TFS-1, TFS-2, TFS-3, TFS-4, TFS-5, and TFS-6, respectively, at the end of release experiment. The order of release for DEX was observed as TFS − 3 > TFS − 1 > TFS − 5 > TFS − 2 > TFS − 4. Statistical analysis of the *in vitro* release results revealed nonsignificant difference (*p* > 0.05) between all the prepared TFS. The slight difference in drug release could be attributed to the difference in structure and composition of surfactants used for preparation of TFS. Additionally, the TFS release mechanism was assessed using regression analysis (best fitting) of myriad kinetics model via DD solver (Microsoft Excel Add-ins). The data, in terms of *R*^2^ values are presented in Table 3. Most fitted model of transferosomal formulations for MLX release was found to be the Korsmeyer-Peppas, having highest *R*^2^ values for MLX release especially for TFS-1, TFS-2, TFS-5, and TFS-6 as compared to the zero order, first order, and Higuchi model. TFS-3 and TF-4 followed first order kinetics for MLX release. The release exponent “*n*” revealed anomalous (non-Fickian) MLX release from TFS-1, TFS-2, TFS-3, to TFS-4 involving both diffusion and erosion processes; whereas, TFS-5 and TFS-6 obeyed quasi-Fickian diffusion for MLX release. Similarly, Korsmeyer-Peppas model was found to be the best fit owing to its highest *R*^2^ values for *in vitro* drug release of DEX from all TFS. The “*n*” value for DEX release from TFS-2, TFS-3, TFS-4, TFS-5, and TFS-6 were found to be < 0.5 displaying release via Fickian diffusion model. However, *n* value for TFS-1 was observed to be 0.602 evidencing their release via non-Fickian diffusion as illustrated in Table 3.

### 3.6. *Ex Vivo* Skin Permeation Studies

**Figure 4:**
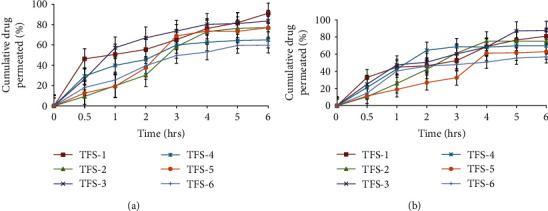
*Ex vivo* skin permeation profiles of MLX (a) and DEX (b) from different transfersomal formulations across excised rat skin (mean ± SD, *n* = 3).

*Ex vivo* skin permeation studies give valuable insights about the *in vivo* behavior of product since they depict absorbable amount of drug. The cumulative amount of drug permeated through hairless rat skin was evaluated using Franz diffusion cell and quantified in terms of percentage drug permeated per unit time. The cumulative amount of MLX permeated at the end of 6 h was 91.12%, 76.99%, 83.47%, 64.89%, 76.93%, and 59. 77% from TFS-1, TFS-2, TFS-3, TFS-4, TFS-5, and TFS-6, respectively, as shown in Figure 4(a). The cumulative amount of DEX permeated at the end of 6 h was 81.01%, 75.25%, 87.8%, 69.74%, 62.95%, and 56.97 from TFS-1, TFS-2, TFS-3, TFS-4, TFS-5, and TFS-6, respectively, as given in Figure 4(b).

### 3.7. Evaluation of Transfersome-Based Gels (TF-G)

#### 3.7.1. Measurement of pH

The pH of drug-loaded plain gel and transfersome-based gels (TF-G1, TF-G3, and TF-G5) was measured in triplicate and results ranged from 5.53-6.85 which are given in Table 4. The accepted pH range for transdermal preparations is in between 4 and 7 [42]. In our investigation, pH of transfersome-based gels was observed neutral (6.13-6.85). Therefore, they were suitable for application and unlikely to induce irritation.

#### 3.7.2. Spreadability

Spreadability is an important property of transdermal formulations from a patient compliance point of view [43]. Spreadability is an important parameter for consistent and easy application of semisolid dosage form. Results obtained from spreadibility studies were recorded as 7.5 ± 0.35 gcm/sec, 7.6 ± 0.26 gcm/sec, 6.8 ± 0.06 gcm/sec, and 5.35 ± 0.02 gcm/sec for TF-G1, TF-G3, TF-G5, and plain gel, respectively, as given in Table 4. These values of spreadibility assure practicability of prepared gels for topical application.

#### 3.7.3. Viscosity Determination

The viscosity of three transfersome-containing gels (TF-G1, TF-G3, and TF-G5) and the plain gel was measured at 30 rpm and 37°C with a viscometer (Brookfield RVDV-II). The viscosity values were 5.8 ± 0.008 Pa.s, 7.4 ± 0.095 Pa.s, 6.8 ± 0.043 Pa.s, and 5.3 ± 0.095 Pa.s for plain gel, TF-G1, TF-G3, and TF-G5, respectively. The results were calculated in triplicate and results are presented in Table 4.

#### 3.7.4. Homogeneity

All transfersomal formulations (TF-G1, TF-G3, and TF-G5) as well all plain gel show good homogeneity with the absence of any lumps or aggregates. It also has been observed that all developed gel formulations were clear and transparent. The results are shown in Table 4.

(Results presented as a mean value ± SD of three measurements, *n* = 3), Pa.s: pascal second.

#### 3.7.5. Release Studies of Transfersome-Based Gels

**Figure 5:**
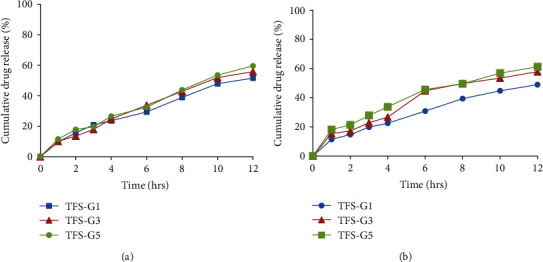
Release profiles of MLX (a) and DEX (b) from transfersome-based gels at pH 7.4.

The percentage of MLX and DEX released after 12 h from different TF-based gels (TF-G1, TF-G3, and TF-G5) at pH 7.4 is represented graphically in Figures 5(a) and 5(b). TF-G1, TF-G3, and TF-G5 showed % cumulative MLX release of 51.54%, 55.80%, and 59.60%, respectively. Likewise, % cumulative release of DEX was found to be 48.98%, 57.88%, and 61.23% for TF-G1, TF-G3, and TF-G5, respectively. These results indicate that the using a carbopol gel as transfersome vehicle, controlled release can be established.

Regarding release kinetics, it was found that all the investigated TF-based gels (TF-G1, TF-G3, and TF-G5 showed a Korsmeyer-Peppas model of the drug release (MLX and DEX) as the correlation of coefficient values were found higher than those obtained from zero order, first order, and Higuchi kinetics as shown in Table 5. It was found for all the investigated TF-based gels that the best fitting was obtained with (*n*) values 0.5 < *n* < 1.0, which indicates anomalous transport of drug release for both drugs.

#### 3.7.6. Skin Irritation Studies

Skin irritation potential of transfersome-based gels (TF-G1) was evaluated and results have been listed in Table 6.

The average erythemal and edema scores were recorded based on severity caused by topical application of formulations, if there is no edema/erythema = 0, slight erythema/edema = 1, moderate erythema/edema = 2, and severe erythema/edema = 3.

#### 3.7.7. *Ex Vivo* Skin Permeation Studies of Transfersome-Based Gel

**Figure 6:**
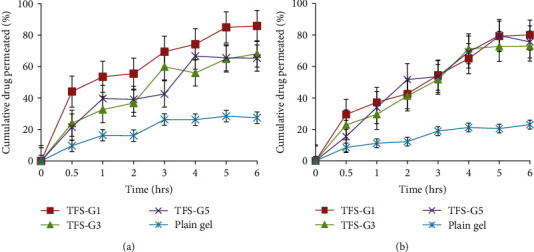
*Ex vivo* skin permeation profiles of MLX (a) and DEX (b) from transfersome-based gels (TF-G1, TF-G3, and TF-G5) and plain drug-loaded gel through hairless rat skin (*n* = 3).

The transfersome-based gels (TF-G1, TF-G3, and TF-G5) and plain drug-loaded gel were characterized *ex vivo* for skin permeation profiles and the results are shown in Figure 6. The total amount of MLX permeated at the end of 6 h was found to be 85.87%, 68.07%, and 65.39% for TF-G1, TF-G3, and TF-G5, respectively; whereas, plain gel demonstrated cumulative MLX release of 27.51%. Similarly, cumulative amount of DEX permeated at the end of 6 h was 79.99%, 72.90%, and 75.65% for TF-G1, TF-G3, and TF-G5, respectively; whereas, plain gel demonstrated cumulative DEX release of 23.05%. In both cases, it was obvious that transfersomal gels had significantly (*p* < 0.05) higher amounts of MLX and DEX compared to plain/nontransfersomal drug-loaded gel.

Kinetic analysis of the permeation data for TF-based gels are presented in Table 7.The results revealed that the permeation of MLX and DEX followed Korsmeyer-Peppas model as indicated by the highest correlation coefficient (*r*) of the model (Table 7). It was found for the investigated TF-based gels that the best fitting was obtained with (*n*) values *n* < 0.5 which indicated Fickian diffusion model. However, *n* value for DEX permeation in TF-G3 and TF-G5 were observed to be 0.516 and 0.522, respectively, demonstrating DEX permeation via non-Fickian diffusion as depicted in Table 7.

The results obtained from *ex vivo* skin permeation studies were fitted in various kinetic models. These model-dependent kinetic evaluation studies of MLX concluded that the best fit model for all prepared transfersome-containing gels was Korsmeyer-Peppas because of maximum regression coefficient (*R*^2^) values as shown in Table 7. Similarly, the most appropriate mathematical model that explains the permeation of DEX across the skin is Korsmeyer-Peppas (Table 6) as all formulations get the highest degree of coefficient, i.e., *R*^2^ = 0.931, *R*^2^ = 0.8924, *R*^2^ = 0.8939, and *R*^2^ = 0.9304 in TF-G1, TF-G3, TF-G-5, and plain gel, respectively.

#### 3.7.8. Anti-Inflammatory Activity Studies of Transfersome-Based Gels

**Figure 7:**
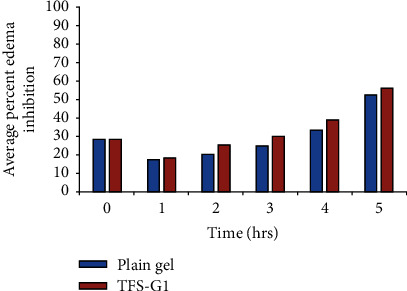
% reduction in edema produced by plain gel and transfersome-based gel (TF-G1) of MLX and DEX in carrageenan-induced paw edema.

The anti-inflammatory and sustaining action of transfersome-based gel (TF-G1) by the carrageenan induced hind paw edema method in Albino rats. The mean percentage inhibition of edema in the TF-G1 and plain nontransfersomal gel containing MLX and DEX as determined by a one way ANOVA differed nonsignificantly with *p* < 0.05. The percent inhibition was found to be greater for TF-G1 gel (50.57% after 5 h) as compared to plain gel which exhibited mean percentage edema inhibition of 47.42%, after 5 hours (Figure 7).

## 4. Discussion

The conventional thin-film hydration (TFH) method was found to be suitable technique for the development of MLX- and DEX-loaded lecithin-based TFS. Previously, this method has shown a promising results for preparation of TFS with desired characteristics [44]. In TFH, the method involved hydration of thin lipid film with PBS (pH 6.4) containing MLX and DEX. The temperature was kept above gel to liquid transition temperature of the surfactants during the evaporation and hydration steps because this is required for the production of niosomes [45]. This technique is usually followed by sonication to allow the formation of vesicles with homogenous size distribution [46]. Six TF formulations were designed using varying concentrations of lecithin and surfactants. Two surfactants (Span 80 and Tween 80), which served as edge activators, were investigated for their impact on physicochemical characteristics of transfersomes. Phospholipids are directing agents for the formation of vesicles. Lecithin was selected as the phospholipid because it has been successfully employed in previous studies for the entrapment of lipophilic drug molecules with good encapsulation capacity and offered controlled drug release [47]. The concentrations of lecithin were kept higher in TFS-1, TFS-3, and TFS-5 compared to TFS-2, TF-4, and TF-6 in transfersomes. Lecithin was employed as lipid in the formulation and can develop lipid bilayer structure. Lecithin is widely reported phospholipid in the literature as being biocompatible, safe, and with excellent pharmaceutical attributes, and its potential in a novel drug delivery system has been investigated and considered as pharmaceutically acceptable [48]. Both surfactants (edge activators), i.e., Tween 80 and Span 80, are biocompatible and nontoxic excipients that are widely used in various forms of pharmaceutical formulations [49]. Further, Span 80 and Tween 80 are capable of imparting greater deformability and permeation as compared to other edge activators [50]. The appropriate ratio of lipid and edge activator provides greater flexibility and deformability and decreases the chances of vesicular rupture when reached in the skin layers [51]. The reported HLB value of Span 80 is 4.3 while the HLB value of Tween 80 is 15 [52]. The interaction between the lipid bilayer and surfactant was considered to be greater for Span 80 and compared to Tween-80. Thus Span 80 provides greater flexibility to TF vesicles than Tween 80 [53]. The results of the current study illustrated that appropriate ratios of lecithin and surfactants can yield TFS loaded with MLX and DEX. Furthermore, to increase the viscosity and to make them more deliverable for transdermal applications, optimized transfersomes TFS-1, TFS-3, and TFS-5 were incorporated into carbopol 940 matrix which played its role as gelling agent.

Drug-excipient interactions were studied before developing the formulation by using FTIR spectroscopy, which is one of the most important analytical tool to investigate the molecular interactions between drugs and the used excipients [54, 55]. Pure MLX spectrum (A-2) showed sharp characteristics peaks at the same absorption bands like that of several independent studies performed earlier [34]. The spectra revealed no major stretching or bending in the position of major absorption bands especially concerning functional groups like amine and carbonyl. Meanwhile, the IR spectrum of the physical mixture of MLX with all excipients did not show any major shift in the regions where characteristic peaks were observed in the pure drug. The FTIR spectra of pure DEX (A-1) confirms its structure by showing the characteristic absorption bands at 1268 cm^−1^, 3390 cm^−1^, 1709 cm^−1^, 1621 cm^−1^, and 900 cm^−1^. The characteristic peaks of DEX were also being found in spectra of physical mixtures (B-1, B-2, B-3, B-4, and B-5) indicating compatibility of ingredients with model drugs. No significant shifts of MLX and DEX peaks were observed when present along with other excipients of the physical mixture indicating compatibility of drugs with transfersomal components.

Entrapping a sufficient amount of a therapeutic agent is one of the most desirable properties of drug delivery system [56]. It is an expression of the amount of drug incorporated into the colloidal drug delivery systems and is normally defined as the percentage of drug bound to carrier relative the total amount of drug [57]. The % entrapment efficiency (%EE) is summarized in Table 2, and it was found that an appreciable amounts of MLX and DEX were entrapped in TFS. Higher %EE of MLX and DEX was achieved with TFS-1, TFS-3, and TFS-5. The higher entrapment efficiency of TFS-1, TFS-3, and TFS-5 might be due to higher concentrations of lecithin used. It is also observed that the TFS showed good drug entrapment when Span 80 and Tween 80 were used separately in formulations. However, the %EE of MLX and DEX reduced when a mixture of Span 80 and Tween 80 was added in formulations (TFS-5 and TFS-6) because the combination of surfactants alters the overall HLB value of the surfactant system leading to weak interaction of drugs with lipid matrix resulting in poor %EE values [58]. It is also reported that the use of the combination of surfactants leads to the formation of mixed micelles which generally show lower drug entrapment capacity [59]. The probable reason for higher entrapment efficiency was the presence of surfactant in optimal concentration, i.e., >20%*w*/*w* [53]. It is documented in previous researches that initially by increasing the quantity of surfactant, entrapment efficiency also increased, but when surfactant concentration reached above 20%*w*/*w*, the complete conversion of vesicles into mixed micelles takes place due to the formation of pores in vesicle membrane [60]. Similarly, another literature study supports the statement in which higher entrapment efficiency was attained using a high concentration of lipids [53]. Some other studies proposed that good entrapment could also be possible by the use of ethanol, methanol, or chloroform for vesicle formation. It can be possible due to an increase in intralamellar distance of vesicle membranes and an increase in fluidity [61]. Thus, all these findings suggest that a suitable concentration of lipid and surfactant ratio results in higher %EE. Greater %EE is considered as an indicator for the successful formulation of the drug delivery system which may have the potential to offer better therapeutic outputs. In addition to this, simple ultracentrifugation method gives inaccurate or overestimated values of entrapment efficiency due to binding of free drug with transfersomal membranes [62]. The higher values of entrapped drugs MLX and DEX might be linked to this factor also.

Particle size is an essential parameter to predict the degree of dissolution, drug penetration to the targeted site, cellular uptake, and therapeutic index of the drug [63]. Furthermore, particle size also affects the %EE, biodistribution, drug release profile, and cellular uptake. Generally, it has been noted that vesicles having a diameter above 600 nm are not able to permeate through the skin along with the entrapped drug. Hence, particle size below 300 nm is regarded ideal for the transdermal delivery of drugs [64]. The analysis report for particle size indicated that the prepared transfersomes having Tween 80 (TFS-1) have the smallest particle size of 248 nm which was in good agreement with vesicles fabricated from Tween 80 reported elsewhere [65]. Moreover, it was well noted that the change of edge activator type from Tween 80 to Span 80 exhibited no significant difference (*p* > 0.05) in the size of particles [65]. The comparable size of vesicles achieved with Tween 80 and Span 80 was probably due to similar amphipathic nonionic inherent characteristics. Transfersome vesicles made by using Tween 80 have greater vesicle size than the vesicles made up of using Span 80. This difference is due to different hydrophile-lipophile balance (HLB) values of the surfactant [66]. Span 80 as an oil-soluble surfactant may interact with the lipophilic chains of the phospholipids in further addition to the presence of lipophilic substances (Dexamethasone), which create a further competition with Span 80 on the phospholipid alkyl chains. The packing of these molecules in the bilayer membrane might be causative of the relatively large vesicle size as compared to Tween 80 [67]. Likewise, the polydispersity index of all six transfersomes was within acceptable value < 0.7 [37]. The term “polydispersity” (or “dispersity” as recommended by IUPAC) is used to describe the degree of nonuniformity of a size distribution of particles [64]. The numerical value of PDI ranges from 0.0 (for a perfectly uniform sample with respect to the particle size) to 1.0 (for a highly polydisperse sample with multiple particle size populations) [68]. The low PDI value of 0.389 indicated a uniform and narrow size distribution as well as homogenous particle distribution throughout the prepared formulations. These results of PDI and size distribution were also similar to the previously reported findings [69].

The zeta potential is the net charge on the surface of particles which is used to evaluate and determine the interaction between the particles. The high negative value of zeta potential makes the TFS suspension electrically stable and helps to avoid micelles or floccules formation and give physical stability to the system [70]. In current study, the values of zeta potential of all three optimized formulations (TFS-1, TFS-3, and TFS-5) were -69.7, -69.6, and -69.8 mV. The formulation (TFS-1) exhibits a highly negative surface charge of -69.7 mV. The higher negative charger on the surface of vesicles produced a repulsive force between the vesicles and made them electrically stable and able to resist any aggregation. This result can be explained by the difference in the ionic nature of the investigated surfactants as well as negative phospholipids found in soya lecithin [71]. This was in agreement with previous findings obtained by El Sayyad et al., [72]. Previous reports mentioned that the electrostatic charge on the vesicle surfaces results from the combination of both lipid and surfactant charge. The higher value of zeta potential is considered desirable for the lipid vesicle formulations stability [73]. It is well documented that nanoparticles with higher negative zeta potential values can resist aggregation and show a good degree of electrical stability [74]. Additionally, as the concentration of the surfactant increased, the net charge of the vesicles gets increased in a previous study conducted by Singh et al. [75] and our findings were consistent with it.

SEM images of TFS-1 and TFS-3 demonstrated a spherical structure of the prepared transfersomes with good vesicle characteristics (Figure 2). Furthermore, SEM images revealed relatively heterogeneous population of transfersomes with broad size distribution. Vesicle morphology is an important parameter for efficient drug entrapment and skin delivery. Hence, in the current study, these uniform-sized vesicles with no or little aggregation were able to entrap large amounts of the drug. In addition to this spherical configuration of vesicles attained in this study supports the better permeation through deeper layers of skin [76]. *In vitro* drug release test was conducted at pH 7.4 to determine release pattern at the body's physiological pH. The assessment of release profile has shown that the drug solutions exhibited 100% release of MLX and DEX at 8 h and 10 h, respectively, which could probably cause frequent dosing of the loaded drugs. MLX and DEX release pattern from designed TFS have displayed a prolonged release pattern with low initial release as compared to pure drug solutions as illustrated in Figures 3(a) and 3(b) . The overall trend of drug release was that Tween 80-based TFS offered maximum drug release (81.87% for MLX and 83.72% for DEX) compared to Span 80-based TFS. This might be due to fact that Tween 80 comprises lipophilic tails with long and unsaturated (C18) moieties facilitating the incorporation of active moieties within the lipid bilayer and resulting in more permeable vesicle membrane [47]. This drug release trend was congruent to previous studies [26]. In addition, the fluid nature of Tween 80 compared to Span 80 comprised for higher MLX and DEX release from TFS. Most of TFS formulations provided best fitting with Korsmeyer-Peppas model indicating diffusion controlled mechanisms of both MLX and DEX release. This property was found to be advantageous approach in terms of reduction in dosing frequency as encountered with plain drug solutions of MLX and DEX [77].

The *ex vivo* permeation studies are considered as predictors for *in vivo* fate of drugs [78]. This study was performed to determine the permeation flux of drugs through intact skin. The results revealed that the maximum percentage of MLX released was 90% from formulation TFS-1 after 6 h. While from the same formulation, 81% of dexamethasone released after 6 h of study. The rate of drug released from prepared formulation was changed for both drugs depending upon the amount of drug entrapped in vesicles. The finding was supported by the results of %EE that was 96% for MLX and 80% for DEX. From this, it can be concluded that the results of *ex vivo* drug release were consistent with that of percentage entrapment efficiency. The higher %EE leads to higher drug release from vesicles into the skin and lower the drug loss from prepared formulation and similar findings have been obtained previously [79]. The current investigation also showed that TFS formed with mixed surfactants, i.e., combination of Span 80 and Tween 80 showed poor *ex vivo* permeation of both drugs while the TFS containing the single surfactant with high lecithin concentration showed the highest cumulative amount of drug permeated. The increased *ex vivo* permeation was also possible due to the flexible nature of vesicles owing to presence of surfactant [11]. The higher release of MLX and DEX from the TFS was attributed due to the nature of prepared vesicles, as drugs were entrapped in nanosized lipid vesicles [20].

After successful characterization of prepared TFS, three TF formulations (TFS-1, TFS-3, and TFS-5) were selected and incorporated into gel matrix using carbopol-940, and gels were coded as TF-G1, TF-G3, and TF-G5) because the viscosity of suspension was too low to use for transdermal drug delivery. Thus, newly developed transfersome-based gel was evaluated for various parameters. Firstly, the pH of all prepared TF-containing gels (TF-G1, TF-G3, and TF-G5) and the plain gel was measured. The results were found to be within an acceptable limit to avoid skin irritation after application [74].

Another, important parameter that must be considered for topically applied formulations was the measurement of spreadability. Spreadability is the result of structural and viscoelastic physiognomies that portray the rigidity, strength along with the relative contributions of elastic, and viscous behavior. The ability of a semisolid formulation to spread on the skin plays a vital role in the administration of a standard medicated formulation and thus in the efficacy of a topical therapy [80]. It is reported earlier and demonstrated in this study that there is a direct correlation between spreadability of a vesicle-loaded pharmaceutical gel and its *in vivo* therapeutic efficacy [81]. The higher values of spreadability indicate the ease of application which leads to an increase in the surface area for drug application and penetration [82]. The spreadability of the plain gel was calculated as 5.35 g.cm/sec while the results of TF-containing gels were higher that ranging from 6.8 to 7.5 g.cm/sec. The high values showed good spreadability by a small amount of shear.

All gels (TF-G1, TF-G3, and TF-G5) revealed similar rheological behavior. In addition to, these gel formulations showed higher values of viscosity which facilitate the transdermal delivery of MLX and DEX [83, 84]. Furthermore, both plain gel and TF-containing gels showed good homogeneity with the absence of any lumps. The good homogeneity indicated that the prepared formulations are uniform in consistency and no appreciable particulate matter was visualized. *In vitro* release studies revealed that at pH 7.4, substantially low amount of MLX and DEX were released from TF-G1, TF-G3, and TF-G5 (Figures 5(a) and 5(b)). The initial burst release gel system could probably be because of the unentrapped drug and desorption of free drug from surface of vesicles. But, later, the release slowed down confirming an extended release pattern. Only 48–60% drug was released in 12 h. This extended release properties of the TF-based gels indicated a reduced dosing frequency of the developed formulations which can further improve patient compliance. *In vitro* release behavior of MLX was analogous to DEX as shown in Figures 5(a) and 5(b). This illustrates the significant lower release of MLX and DEX from TF-based gels compared to the release from the pure TFS. The higher viscosity of TF-based gels due to the lower water content and presence of carbopol 940 (gelling agent) could lead to slow diffusion of the entrapped drug [85]. The another reason for low drug release from TF-based gels is due to matrix release phenomenon of drug from Carbopol-940 gel after the diffusion controlled drug release from TFS [19]. Various release kinetic models applied are given in Table 5 and *R*^2^ values revealed that TF-based gels followed Korsmeyer-Peppas model since they showed highest *R*^2^ value. Diffusion exponent (*n*) values revealed that MLX and DEX followed non-Fickian diffusion (anomalous) based release from TF-based gels as shown in Table 5. It could be concluded from the studying of release kinetics that MLX and DEX diffusion from gel network structure and partitioning through TF vesicles are responsible for continuous delivery of drugs. Non-Fickian diffusion is also based on swelling of vesicle followed by release of the drug. Since hydrophilic drugs like DEX is incorporated in aqueous core and it cannot diffuses out of the lipid bilayer itself, therefore swelling might help in opening channels for release of DEX [40].

Plain gel and TF-G preparations were subjected to *ex vivo* skin permeation studies. Cumulative amount of MLX and DEX released from each formulation was calculated. The results indicated that higher amounts of MLX and DEX permeated from transfersome-containing gel as compared to plain gel. Only 26% of MLX and 22% of DEX were permeated through the skin from plain gel; whereas, 85% of MLX and 78% of DEX were released from TF-G formulations over 6 h of study revealing statistical significant difference (*p* < 0.05). The higher drug release from optimized TFS based could be due to the nature of the formulations as drugs were present in nanosized lipid bilayer structures. Furthermore, the entrapment of drugs in TF vesicles also facilitates the permeation of drugs through intact skin due to the unique ability of drug-containing vesicles. The smaller particle size of TFS that are incorporated inside the gel is also a reason for improved skin permeation as compared to plain gel. Likewise, the value of *n* was calculated and the obtained results revealed that *n* is less than equal to 0.5 for all formulations. Likewise, after studying the kinetic models it is evident in Tables 5 and 6, the values of release rate constant (*R*^2^) and regression coefficient (*n*) confirm that the gel formulations followed Korsmeyer-Peppas which is also known as super case II. The super case II is mostly observed when the chain relaxation process is slower than diffusion and the process of drug released is controlled with the help of diffusion only [86]. The results of *n* value indicated that all formulations govern the mechanism of penetration known as the Fickian diffusion method which means drug molecules diffused through chemical diffusion gradient [87].

## 5. Conclusion

MLX- and DEX-loaded TFS and corresponding carbopol-940 gels were successfully developed and optimized for transdermal applications. TFS were prepared using thin-film hydration technique. The concentration of lecithin significantly affected the entrapment efficiency values. On the other hand, increase in concentration of surfactant decreased the entrapment efficiency values. The size of transfersomal vesicles was influenced by concentrations of lecithin and surfactants used. Small TFS were obtained at higher concentration of lecithin and lower concentration of surfactants. Statistical significant differences were observed in vesicle size (*p* < 0.05) depicting substantial effects of lecithin and surfactant on size of TFS. The release profiles of TFS as well as TF-based gels exhibited improved and relatively extended patterns compared to drug solutions. TF-based gels showed improved permeation (*p* < 0.05) of MLX and DEX compared to plain (nontransfersomal) drug-loaded gel. Over all, TF-based gels of MLX and DEX could be considered as an alternative delivery approach for enhanced skin permeation.

## Figures and Tables

**Table 1:** Composition of transfersomes.

Materials used	Formulation codes					
	TFS-1	TFS-2	TFS-3	TFS-4	TFS-5	TFS-6
Lecithin (mg)	150	90	150	90	150	90
Span 80 (mg)	90	150	—	—	45	75
Tween 80 (mg)	—	—	90	150	45	75
MLX (mg)	50	50	50	50	50	50
DEX (mg)	25	25	25	25	25	25
Methanol: chloroform	3 : 1	3 : 1	3 : 1	3 : 1	3 : 1	3 : 1
Phosphate buffer pH 6.4 (mL)	20	20	20	20	20	20

**Table 2:** % entrapment efficiency, vesicle size, PDI, and zeta potential of transfersomes.

Transfersomes	% entrapment efficiency		Vesical size (nm ± SD)	PDI values	Zeta potential (mV ± SD)
	MLX	DEX			
TFS-1	96 ± 4	80 ± 2	248 ± 3	0.389 ± 0.03	−63.7 ± 4
TFS-2	82 ± 2	75 ± 8	272 ± 5	0.489 ± 0.04	−69.5 ± 5
TFS-3	89 ± 5	81 ± 5	256 ± 2	0.368 ± 0.05	−62.6 ± 2
TFS-4	63 ± 3	77 ± 8	273 ± 4	0.415 ± 0.02	−68.7 ± 6
TFS-5	81 ± 6	79 ± 4	251 ± 6	0.329 ± 0.03	−63.8 ± 2
TFS-6	67 ± 4	48 ± 3	261 ± 4	0.526 ± 0.05	−66.7 ± 3

**Table 3:** Release kinetics of MLX and DEX from transfersomes.

Formulation codes	Zero order		First order		Higuchi model		Korsmeyer-Peppas model	
	*R* ^2^	*K*_0_	*R* ^2^	*K*_1_	*R* ^2^	*K*_*H*_	*R* ^2^	*n*
TFS-1 (MLX)	0.8605	7.287	0.9911	0.124	0.9777	21.095	0.9914	0.604
TFS-2 (MLX)	0.7884	7.563	0.9667	0.135	0.9875	22.079	0.9896	0.538
TFS-3 (MLX)	0.7899	8.362	0.9868	0.164	0.9811	24.401	0.9841	0.546
TFS-4 (MLX)	0.8920	6.895	0.9732	0.111	0.9319	19.774	0.9654	0.678
TFS-5 (MLX)	0.7193	7.617	0.9245	0.138	0.9892	22.356	0.9895	0.485
TFS-6 (MLX)	0.7399	7.644	0.9367	0.139	0.9938	22.404	0.9938	0.496
TFS-1 (DEX)	0.8605	7.287	0.9922	0.146	0.9763	23.124	0.9894	0.602
TFS-2 (DEX)	0.7884	7.563	0.9601	0.155	0.9845	23.538	0.9847	0.491
TFS-3 (DEX)	0.7899	8.362	0.9723	0.182	0.9909	25.411	0.9925	0.469
TFS-4 (DEX)	0.8920	6.895	0.9096	0.131	0.9934	21.684	0.9963	0.459
TFS-5 (DEX)	0.7193	7.617	0.9390	0.151	0.9902	23.433	0.9903	0.490
TFS-6 (DEX)	0.7399	7.644	0.9488	0.142	0.9991	22.601	0.9994	0.487

**Table 4:** pH, spreadibility, viscosity, and homogeneity of transfersome-based gels.

Formulation codes	pH	Spreadability (g.cm/s)	Viscosity (Pa.s)	Homogeneity
Plain gel	5.53 ± 0.13	5.35 ± 0.02	5.8 ± 0.008	Good
TF-G1	6.85 ± 0.03	7.5 ± 0.35	7.4 ± 0.095	Good
TF-G3	6.78 ± 0.03	7.6 ± 0.26	6.8 ± 0.043	Good
TF-G5	6.13 ± 0.06	6.8 ± 0.06	5.3 ± 0.095	Good

**Table 5:** Release kinetics of MLX and DEX from transfersome-based gels.

Formulation codes	Zero order		First order		Higuchi model		Korsmeyer-Peppas model	
	*R* ^2^	*K*_0_	*R* ^2^	*K*_1_	*R* ^2^	*K*_*H*_	*R* ^2^	*n*
TF-G1 (MLX)	0.9326	4.768	0.9784	0.064	0.9554	13.639	0.9939	0.696
TF-G3 (MLX)	0.9650	5.145	0.9946	0.070	0.9301	14.593	0.9953	0.773
TF-G5 (MLX)	0.9513	5.371	0.9835	0.075	0.9404	15.289	0.9923	0.739
TF-G1 (DEX)	0.9116	4.617	0.9711	0.061	0.9684	13.266	0.9958	0.658
TF-G3 (DEX)	0.8668	5.673	0.9595	0.083	0.9567	16.371	0.9754	0.626
TF-G5 (DEX)	0.7987	6.028	0.9409	0.092	0.9910	17.579	0.9936	0.543

**Table 6:** Mean erythemal scores measured at the end of 24, 48, and 72 h.

Formulations	Erythema scores (*n* = 3)		
	24 h	48 h	72 h
Simple gel (negative control group I)	0	0	0
Solution of MLX and DEX	0	0	1
0.8%*v*/*v* formalin solution in water	2	3	3
TF-G1 (transfersome-based gel)	0	0	0

**Table 7:** Kinetic analysis of permeation data of transfersome-based gels.

Formulation codes	Zero order		First order		Higuchi model		Korsmeyer-Peppas model	
	*R* ^2^	*K*_0_	*R* ^2^	*K*_1_	*R* ^2^	*K*_*H*_	*R* ^2^	*n*
TF-G1 (MLX)	0.3363	17.883	0.8050	0.462	0.8905	38.711	0.9861	0.288
TF-G3 (MLX)	0.6658	13.748	0.8749	0.237	0.9621	29.260	0.9692	0.432
TF-G5 (MLX)	0.6256	13.626	0.8268	0.233	0.9271	29.031	0.9371	0.418
TF-G1 (DEX)	0.7417	15.748	0.9143	0.298	0.9788	33.302	0.9806	0.463
TF-G3 (DEX)	0.7932	14.957	0.9532	0.270	0.9774	31.507	0.9778	0.516
TF-G5 (DEX)	0.7883	15.724	0.9642	0.301	0.9706	33.147	0.9712	0.522

## Data Availability

Authors declare that all the data supporting the findings of this study are included in the article.
